# The *Parapoynx Stagnalis* Nucleopolyhedrovirus (PastNPV), a Divergent Member of the *Alphabaculovirus* Group I Clade, Encodes a Homolog of Ran GTPase

**DOI:** 10.3390/v14102289

**Published:** 2022-10-18

**Authors:** Robert L. Harrison, Daniel L. Rowley

**Affiliations:** Invasive Insect Biocontrol and Behavior Laboratory, Beltsville Agricultural Research Center, USDA Agricultural Research Service, Beltsville, MD 20705, USA

**Keywords:** baculovirus, *Alphabaculovirus*, *Parapoynx stagnalis*, Ran GTPase, enhancin

## Abstract

We report the analysis of the genome of a novel *Alphabaculovirus*, *Parapoynx stagnalis* nucleopolyhedrovirus isolate 473 (PastNPV-473), from cadavers of the rice case bearer, *Parapoynx stagnalis* Zeller (Lepidoptera: Crambidae), collected in rice fields in Kerala, India. High-throughput sequencing of DNA from PastNPV occlusion bodies and assembly of the data yielded a circular genome-length contig of 114,833 bp with 126 annotated opening reading frames (ORFs) and six homologous regions (*hr*s). Phylogenetic inference based on baculovirus core gene amino acid sequence alignments indicated that PastNPV is a member of the group I clade of viruses in genus *Alphabaculovirus*, but different phylogenetic methods yielded different results with respect to the placement of PastNPV and four similarly divergent alphabaculoviruses in the group I clade. Branch lengths and Kimura-2-parameter pairwise nucleotide distances indicated that PastNPV-473 cannot be classified in any of the currently listed species in genus *Alphabaculovirus*. A unique feature of the PastNPV genome was the presence of an ORF encoding a homolog of Ran GTPase, a regulator of nucleocytoplasmic trafficking. PastNPV appears to have acquired a homolog of Ran relatively recently from a lepidopteran host via horizontal gene transfer.

## 1. Introduction

Baculoviruses (family *Baculoviridae* [1]) have two features that continue to inspire their development as biopesticides: their high virulence against insect pests, and their lack of toxicity against non-host organisms and the environment [2]. The latter feature, however, is accompanied by a narrow host range usually consisting of only one or a few related species of pests against which any given baculovirus is optimally active. For this reason, the identification and characterization of new baculoviruses is necessary to identify isolates with insecticidal activity against current pests of interest as well as potential or emerging pests.

Species of *Baculoviridae* are classified into four genera [3]. Viruses from two of these genera, *Alphabaculovirus* and *Betabaculovirus*, infect larvae of Lepidoptera, and some have been formulated for use against lepidopteran pests of agriculture [4,5]. These viruses have large (80–180 kbp) double-stranded DNA genomes that are replicated and packaged into rod-shaped nucleocapsids in the nucleus of infected cells. Initially, these nucleocapsids exit the nucleus and bud from the plasma membrane, forming enveloped virions that spread infection to other cells and tissues within infected larvae. Later during the replication cycle, progeny nucleocapsids are enveloped within the nucleus and assembled into paracrystalline occlusion bodies (OBs). *Alphabaculovirus* OBs are polyhedral in shape with a typical diameter of 0.5–5 µm, and contain several enveloped virions, often with multiple nucleocapsids bundled within a single virion. Betabaculovirus OBs are smaller (0.12 µm wide × 0.50 µm long), ovocylindrical in shape, and contain a single virion with a single nucleocapsid. OBs can be harvested from dead host larvae and applied in the field to trigger epizootics in pest populations. The OB matrix, consisting of the polyhedrin protein (or granulin, for betabaculovirus OBs), provides a degree of protection to the occluded virions against environmental degradation, but readily dissolves in the alkaline environment of the host larval midgut. The occluded virions liberated from the dissolving OBs initiate infection of larval midgut epithelial cells. Infection spreads to other tissues in the larvae, causing nuclear polyhedrosis disease characterized by cell lysis, larval death, and weakening and rupture of the larval cuticle. The OBs released from dead larvae serve to spread the infection to other larvae feeding on virus-contaminated foliage.

In 1977, researchers of the College of Agriculture, Vellayani, Kerala in India observed and collected dead, occlusion-containing larvae of the rice case bearer, *Parapoynx stagnalis* Zeller (Lepidoptera: Crambidae), from local rice fields [6]. The rice case bearer, also referred to as the rice caseworm with the synonym *Nymphula depunctalis* Guenée, is a widespread pest of rice [7,8]; https://www.cabi.org/isc/datasheet/44593 (accessed on 29 August 2022)). Feeding occlusions from the collected cadavers to *P. stagnalis* larvae caused symptoms of nuclear polyhedrosis leading to larval mortality. Infection of the larvae was characterized by the appearance of polyhedral OBs which contained rod-shaped virions and dissolved in alkaline solutions [9]. The properties and appearance of the OBs and symptoms of the disease they caused resembled those of an *Alphabaculovirus*.

A sample of this virus was sent to the Insect Biocontrol Laboratory in Beltsville, MD, USA in 1979. To identify and characterize this virus, we determined and analyzed its complete genome sequence. Our results indicate that this virus, henceforth named *Parapoynx stagnalis* nucleopolyhedrovirus (PastNPV), is a new member of the group I clade of genus *Alphabaculovirus*.

## 2. Materials and Methods

### 2.1. Virus Sample

A PastNPV occlusion body (OB) sample was received from Abraham Jacob at the Kerala Agricultural University College of Agriculture in Vellayani, India, on 29 January 1979. The virus was designated isolate 473 and deposited in the USDA-ARS insect virus collection in Beltsville, MD.

### 2.2. DNA Isolation and Sequencing

OBs were pelleted from 500 µL of the PastNPV sample by microcentrifugation at 9168× *g* for 2 min. The supernatant was removed and the pelleted material was re-suspended in 0.1 M Na_2_CO_3_. The suspension was incubated for 30 min at the benchtop, then neutralized by addition of Tris-HCl pH 7.5 solution to a final volume of 0.1 M. Sodium dodecyl sulfate (10%) and proteinase K (Thermo Fisher Scientific, Waltham, MA, USA, catalog #AM2546) were added to final concentrations of 0.25% *v/v* and 500 µg/mL, respectively, and the suspension was incubated at 55 °C for 1 hr. DNA was extracted by mixing the dissolved OB solution with an equal volume of 25:24:1 phenol: chloroform: isoamyl alcohol saturated with 10 mM Tris-HCl pH 8/1 mM EDTA and separating aqueous and organic phases by centrifugation. The aqueous phase was transferred to a fresh Eppendorf tube and the DNA was precipitated with ethanol at −30 °C overnight. Precipitated DNA was pelleted by centrifugation, re-suspended in deionized distilled H_2_O, and quantified with Quant-iT ™ PicoGreen ™ dsDNA Assay Kit (Thermo Fisher Scientific, #P7589) and a QuantiFluor ™-ST Fluorometer (Promega, Madison, WI, USA). The procedure yielded 197 ng of DNA.

A library for sequencing the DNA sample on an Illumina MiSeq system was constructed from 100 ng of the sample as previously described [10]. Reads were assembled into a contigs and positions with variants were identified using Lasergene NGen v. 16 (DNAStar, Madison, WI, USA).

The first nucleotide of the polyhedrin (*polh*) open reading frame (ORF) was set as the first nucleotide in the genome sequence, and downstream annotated ORFs were numbered accordingly. The annotated PastNPV-473 genome sequence has been deposited in GenBank with the accession number ON704650.

### 2.3. Genome Annotation

ORFs were initially identified using the NCBI Open Reading Frame Finder (https://www.ncbi.nlm.nih.gov/orffinder/ (accessed on 3 March 2022)). ORFs were annotated if they encoded amino acid sequences with significant sequence similarity with other baculovirus ORFs or genes from other sources. ORFs of at least 50 codons with no sequence similarity detected by BLASTp were also annotated if (a) they did not occur within an *hr*; (b) they did not overlap an *hr* or larger ORF by >75 bp, and (c) they were predicted by both FGENESV (http://linux1.softberry.com/berry.phtml (accessed on 3 March 2022)) and GeneMarkS [11] to be protein-encoding sequences. ORFs with no match in a BLASTp query were used in HMM-HMM queries with HHpred [12].

A search for baculovirus homologous regions *(hr*s; [13]) in the PastNPV-473 genome sequence was conducted with Tandem Repeats Finder [14] and the pattern-finding function of Lasergene GeneQuest 17 (DNASTAR). Unit repeats were aligned with MUSCLE [15] as implemented in Lasergene MegAlign Pro 17 (DNASTAR) with default parameters.

### 2.4. Phylogeny

Baculovirus core gene amino acid alignments were downloaded from the *Baculoviridae* chapter of the ICTV Online Report (https://ictv.global/report/chapter/baculoviridae/baculoviridae/resources (accessed on 7 April 2022)) and re-aligned with core gene amino acid sequences from PastNPV-473 and selected other baculoviruses (Table S1) with MAFFT [16] or MUSCLE [15] as implemented in Lasergene MegAlign v. 17. The core gene alignments, which contained sequences from 97 baculoviruses, were concatenated with BioEdit 7.2.6 [17] and phylogeny was inferred by maximum likelihood (ML) using RAxML [18] from the concatenated core gene alignments using the Le and Gascuel (LG) substitution matrix [19] with variable rates among sites, empirical amino acid frequencies, and 100 rapid bootstrap replicates. Phylogenies were also inferred by minimum evolution (ME) in MEGA XI [20] with distances calculated using the JTT-based matrix method [21] with a gamma shape parameter of 0.79 and 500 bootstrap replicates.

PastNPV-473 ORFs 69 (*enhancin*) and ORF126 were aligned with other baculovirus homologs (Table S1) by MUSCLE and phylogenies inferred with RAxML as described above for the core gene phylogeny.

### 2.5. ORF Synteny and Pairwise Distance Estimation

Synteny of ORFs between PastNPV-473 and selected alphabaculoviruses was assessed with gene-parity plots [22]. Pairwise nucleotide distances between *Alphabaculovirus* partial *lef-8*, *lef-9*, and *polh* sequences were estimated using the Kimura-2-parameter substitution matrix with gamma parameters estimated using MEGA XI [20,23].

## 3. Results

### 3.1. Characteristics of the PastNPV-473 Genome

**Figure 1:**
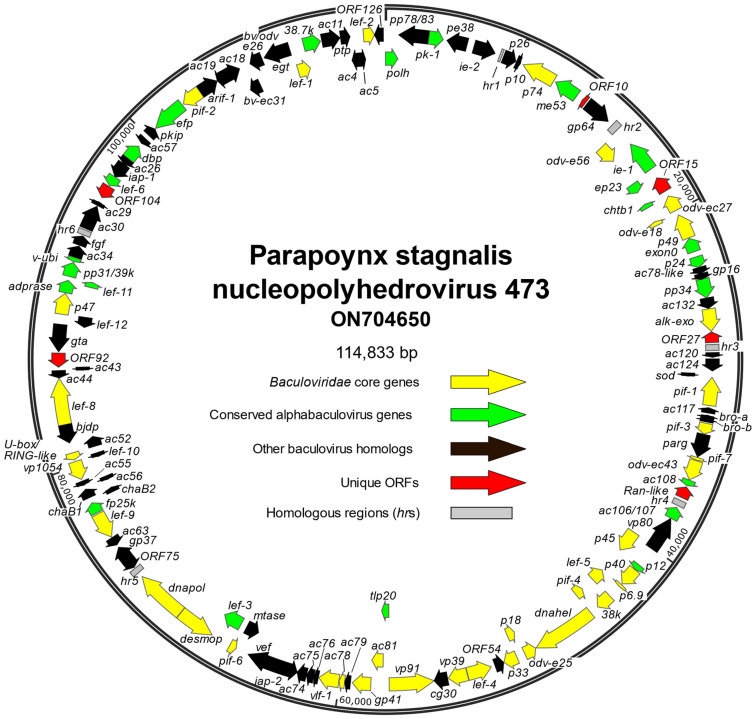
Physical map of the genome of *Parapoynx stagnalis* nucleopolyhedrovirus (PastNPV) isolate 473. The position and orientation of open reading frames (ORFs) are represented by arrows, which are color-coded to indicate ORFs corresponding to core genes of family *Baculoviridae* (yellow), ORFs that are conserved among viruses of genus *Alphabaculovirus* (green), ORFs with homologs in a subset of baculoviruses (black), or ORFs unique to PastNPV-473 (red). ORFs are designated by the names with which they are referenced in the literature, or by a number corresponding to their annotation in the PastNPV-473 genome. Gray boxes correspond to homologous regions (*hr*s).

Sequencing of the PastNPV-473 sample yielded 160,135 reads with an average length of 150 nt that assembled into a 114,833 bp circular contig with an average coverage of 211X and a nucleotide distribution of 34.17% GC. One hundred and twenty-six ORFs were annotated, including the 38 ORFs that constitute the core gene set for family *Baculoviridae* [24,25] (Figure 1, Table S2).

**Figure 2:**
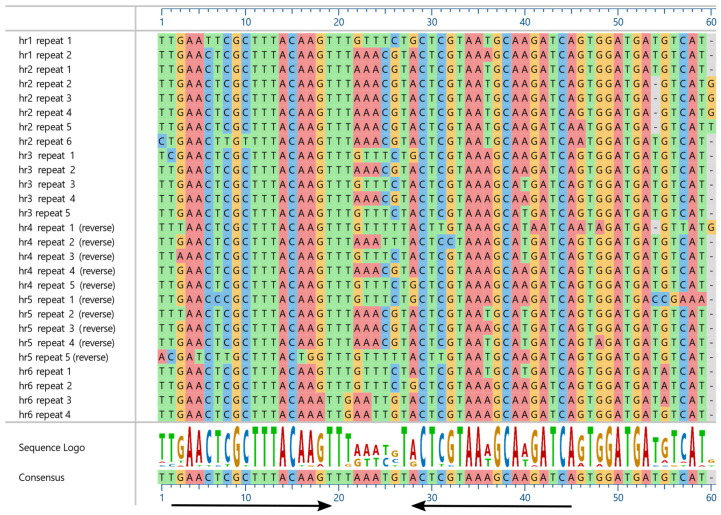
Alignment of the conserved repeats in PastNPV-473 *hr*s. The four bases in the sequences are shaded with different colors to help visualize sequence identity. Repeats present in an orientation opposite to that of the *hr1* repeats are indicated (reverse), as are the consensus sequence and a sequence logo displaying the relative frequency of nucleotide residues at each position [26]. Arrows denote an imperfect palindrome within the unit repeats.

In addition, six regions with conserved repeat sequences were identified as *hr*s. These *hr*s consist of 2–6 59-bp conserved unit sequences containing inverted imperfect repeats, with the consensus sequence 5′-TTGAACTCGCTTTACAAGTTTAAATGTACTCGTAAAGCAAGATCAGTGGATGATGTCA-3′ (Figure 2).

Variant positions were documented in the assembly, including 116 single-nucleotide polymorphisms (SNPs) and 16 insertions and deletions (indels) occurring at frequencies >10% (Table S3). Of the 116 SNPs, 30 of the positions occur in *hr*s or intergenic regions, while the remainder occur in annotated ORFs, resulting in synonymous (40 SNPs) and nonsynonymous (46 SNPs) changes. Of the 16 indels, 12 occur in *hrs* or intergenic regions. The remaining four indels occurred in annotated ORFs, with three maintaining the reading frame and one indel causing a frame-shift.

### 3.2. Comparisons of PastNPV-473 with Other Viruses

BLASTx queries with PastNPV-473 ORFs yielded top matches predominantly with group I alphabaculoviruses, including Lonomia obliqua multiple nucleopolyhedrovirus (LoobMNPV [27], 29 ORFs), Bombyx mori nucleopolyhedrovirus (BmNPV [28]; 20 ORFs), Rachiplusia ou multiple nucleopolyhedrovirus (RoMNPV [29], 11 ORFs), Catopsilia pomona nucleopolyhedrovirus (CapoNPV [30], 11 ORFs), Thysanoplusia orichalcea nucleopolyhedrovirus (ThorNPV [31], 10 ORFs), Autographa californica multiple nucleopolyhedrovirus ([32], 8 ORFs), Maruca vitrata nucleopolyhedrovirus (MaviNPV [33], 6 ORFs), *Plutella xylostella* multiple nucleopolyhedrovirus (PlxyMNPV [34], 6 ORFs), and Troides aeacus nucleopolyhedrovirus (TraeNPV [35], 4 ORFs). Another 11 PastNPV ORFs exhibited matches with one or two ORFs each from eleven other alphabaculoviruses (Table S2).

**Figure 3:**
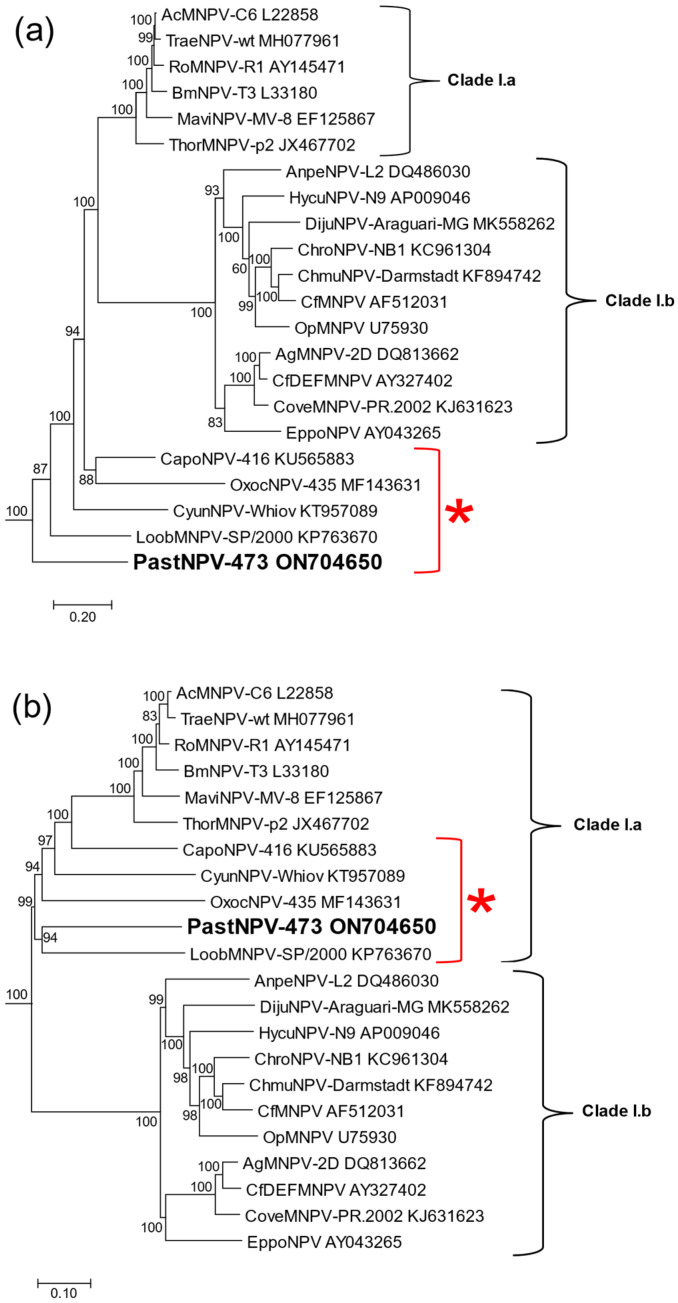
Phylogeny of alphabaculoviruses in the group I clade. (**a**) A subtree of the group I alphabaculoviruses, derived from a phylograms inferred from concatenated MAFFT alignments of baculovirus core gene amino acid sequences by maximum likelihood. (**b**) A subtree of group I alphabaculoviruses derived from a phylogram inferred from MUSCLE alignments of baculovirus core gene amino acid sequences inferred by minimum evolution. Both subtrees show the level of bootstrap support for each branch. Abbreviations and Genbank accession numbers of viruses in the tree are provided; the viruses used in this analysis are listed in Table S1. Clade I.a and I.b are indicated by brackets, and a red bracket and asterisk denotes a group of five alphabaculoviruses with variable placement in the (**a**,**b**) group I subtrees. PastNPV-473 is indicated in bold type.

Phylogenetic inference based on baculovirus concatenated alignments of core gene amino acid sequences placed PastNPV-473 in the group I clade of viruses in genus *Alphabaculovirus* with strong bootstrap support (Figure 3). The assignment of PastNPV as a group I *Alphabaculovirus* is further supported by the occurrence of PastNPV-473 ORF11, a homolog for the *Alphabaculovirus* gene *gp64* (Table S2). The *gp64* gene encodes a budded virus envelope protein (GP64) and is the distinguishing feature of clade I alphabaculoviruses [36]. A previous analysis divided all group I alphabaculoviruses into two sub-clades designated clade I.a and clade I.b [37]. However, in a phylogram produced with the same procedures (ML with the LG substitution matrix inferred from MAFFT-aligned core gene sequences, Figure 3a), PastNPV and four other alphabaculoviruses—CapoNPV, Cyclophragma undans nucleopolyhedrovirus (CyunNPV; [38]), Oxyplax ochracea nucleopolyhedrovirus (OxocNPV; [39]), and LoobMNPV—were not placed in either clade I.a or clade I.b. Instead, PastNPV-473 and the other four viruses occurred on branches lying outside of these two subclades, with PastNPV-473 occupying a position that is basal to the rest of the viruses in the group I clade. A different phylogram produced by ME with JTT-based distances from MUSCLE-aligned core gene sequences placed PastNPV and these four viruses in clade I.a (Figure 3b), while a second ML phylogram based on the same MUSCLE alignments (Figure S1) exhibited the same topology as the ML tree based on the MAFFT alignments (Figure 3a).

The branch lengths in all three phylograms suggested that PastNPV represents a new species in genus *Alphabaculovirus*. The ranges of Kimura-2-parameter pairwise nucleotide distances in alignments of *lef-8*, *lef-9*, and *polh* between PastNPV-473 and alphabaculoviruses were estimated as 0.860–2.092 (*lef-8*), 0.400–2.330 (*lef-9*), and 0.468–1.205 (*polh*) substitutions/site (Table S4). These values were well above the 0.050 substitutions/site demarcation criterion defined for baculovirus species [23,40], further indicating that PastNPV-473 should be classified into a new *Alphabaculovirus* species.

**Figure 4:**
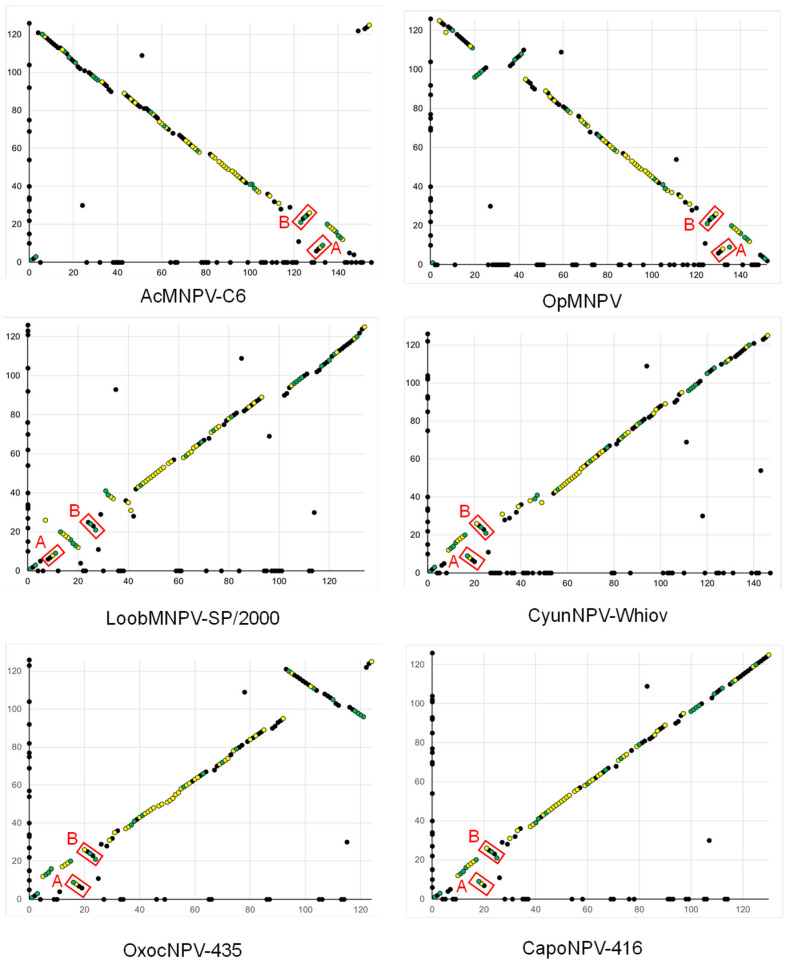
Gene-parity plots comparing the ORF content and order of PastNPV-473 (*y*-axis) with six other group I alphabaculviruses (*x*-axis). Points correspond to individual ORFs. ORFs occurring only in one of the two genomes being compared are plotted directly on the axis corresponding to the virus containing the ORF. Points are color-coded to indicate baculovirus core genes (yellow) and genes conserved among *Alphabaculovirus* genomes (green). Two clusters of ORFs that are inverted in PastNPV-473 relative to ORFs of the other viruses in the plots are indicated by red boxes (A and B).

In gene-parity plots comparing PastNPV to a clade I.a virus (AcMNPV), a clade I.b virus (OpMNPV), and the four viruses with variable placement in the group I subtree (LoobMNPV, CapoNPV, CyunNPV, and OxocNPV), a small number of ORFs in the region of the *polh* (polyhedrin) ORF were found to occur in an orientation in PastNPV that is opposite to their orientation in AcMNPV and OpMNPV, but in the same orientation in the other four viruses (Figure 4). This trend has also been reported in prior comparisons between clade I alphabaculoviruses [30,38,39], indicating that ORF orientation relative to *polh* is a feature that distinguishes the group of alphabaculoviruses including PastNPV, LoobMNPV, CapoNPV, CyunNPV, and OxocNPV from other clade I.a and I.b alphabaculoviruses. In addition, two inversions are evident in the PastNPV-473 genome: one inversion (red box A in Figure 4) encompasses PastNPV ORFs 6 (*p26*) to 9 (*me53*), and the second (red box B) encompasses PastNPV ORFs 21 (*p24 capsid*) to 26 (*alk-exo*). The ORFs in the A inversion are in the same orientation in LoobMNPV and PastNPV, suggesting that this inversion was present in both PastNPV and LoobMNPV.

A BLASTn query with the consensus *hr* repeat sequence of PastNPV-473 resulted in matches to *hr* sequences from isolates of clade I.a viruses, especially AcMNPV and BmNPV, with e-values ranging from 1 × 10^−4^ to 2 × 10^−9^. An alignment of the *hr* consensus sequences for the exemplar isolates of BmNPV and AcMNPV with the *hr* consensus sequence of PastNPV-473 and OxocNPV-435 revealed a conserved imperfect palindromic sequence with the *Eco*R I endonuclease site of the AcMNPV *hr* repeats at its center [41] (Figure S2). The *hr* sequences from different viruses usually share relatively little sequence similarity, but similarity has been observed among *hr*s of related viruses of some clades [42].

### 3.3. ORF Content of PastNPV-473

In addition to homologs for the 38 core genes of family *Baculoviridae*, the PastNPV-473 genome contains homologs for the 26 ORFs reported to be conserved among *Alphabaculovirus* genomes by Garavaglia et al. [24] (Table S2). The PastNPV-473 sequence is missing ORFs for the viral cathepsin and chitinase genes. These ORFs are present in most alphabaculoviruses and several betabaculoviruses, and have been shown to play a role in liquefaction of the host internal anatomy and weakening of the host larval cuticle [43]. While LoobMNPV is also missing these two genes, cathepsin and chitinase ORFs are present in CapoNPV, CyunNPV, and OxocNPV, located between the pkip and dbp ORFs. PastNPV has a homolog of AcMNPV ORF *ac57* present at this this location in place of cathepsin and chitinase ORFs (Table S3).

Of the remaining 62 ORFs, 51 are homologs of ORFs found in AcMNPV. One ORF, ORF22, appears to be a second, divergent copy of core gene ac78. Although a BLASTx query with the ORF22 sequence did not yield any matches, an HHpred query produced UniProt and Pfam matches with AC78 at a probability of >92% with residues 6–59. The remaining 10 ORFs are a mixture of homologs found in other baculoviruses and ORFs with no similarity to previously reported baculovirus sequences.

#### 3.3.1. ORFs with Homologs in Other Alphabaculoviruses

PastNPV ORF54 encodes a putative polypeptide with BLAST matches to ORFs from group I and II alphabaculoviruses that contain the conserved AAA+ ATPase module. The AAA+ (ATPases Associated with a variety of cellular Activities) superfamily of ATPases play a role in a wide range of cellular functions [44]. The top BLAST match for ORF54 is with ORF152 of the group II *Alphabaculovirus* Leucania separata nucleopolyhedrovirus (LeseNPV; [45]), with 41% sequence identity. However, the PastNPV homolog encodes a 170-amino acid polypeptide, while the other baculovirus AAA+ ATPase homologs range from 307 to 448 amino acids with an average of 374 amino acids. AAA+ ATPase active sites are formed by two conserved motifs, Walker A (G(x)4GKT) and Walker B (hhhhDE, where h denotes hydrophobic amino acids) [46]. While both motifs are present in other *Alphabaculovirus* AAA+ homologs, the PastNPV ORF54 sequence only possesses the Walker A motif and thus is unlikely to encode a functional AAA+ ATPase.

**Figure 5:**
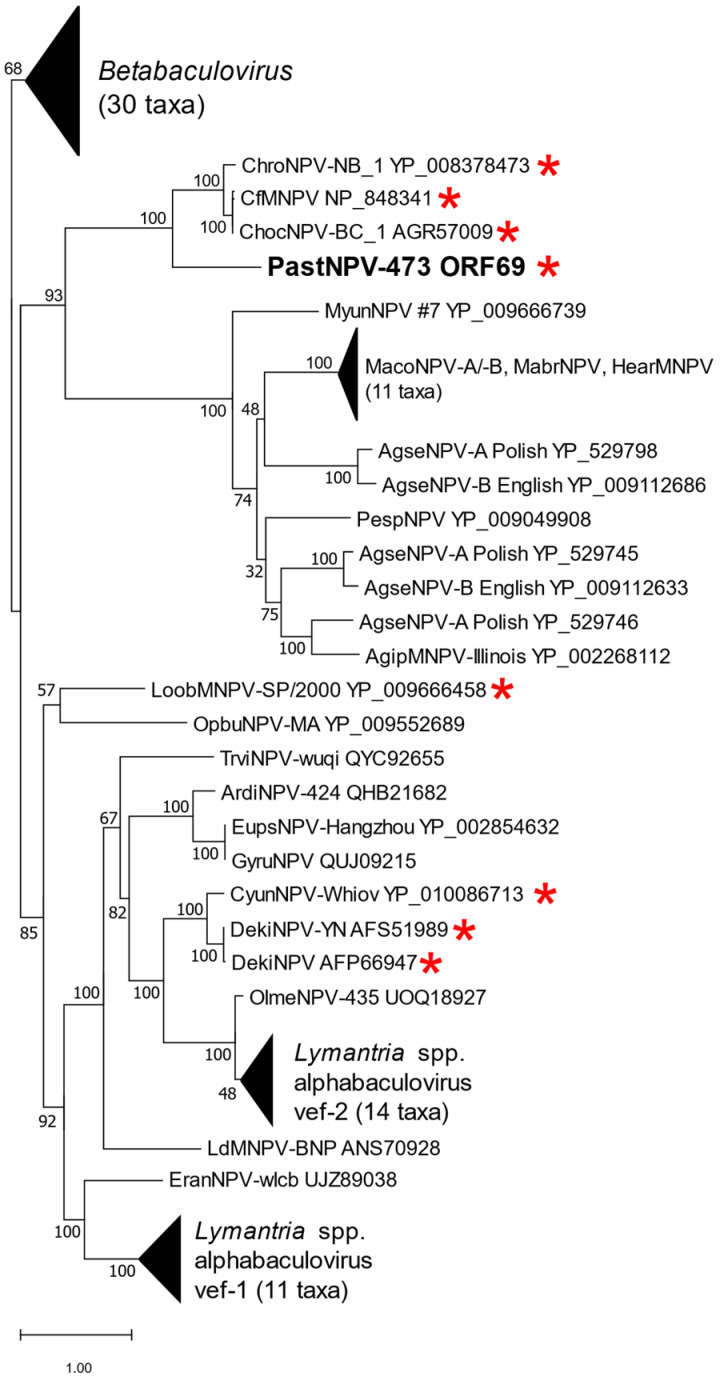
Phylogeny of PastNPV-473 ORF69 and other enhancin (viral enhancing factor; vef) amino acid sequences from baculoviruses. A maximum likelihood phylogeny inferred from an alignment of enhancin homologs is shown, with betabaculovirus enhancin sequences as an outgroup and bootstrap support for the branches. Abbreviations and Genbank accession numbers of viruses in the tree are provided; the viruses used in this analysis are listed in Table S1. PastNPV-473 ORF69 is indicated in bold type, and group I *Alphabaculovirus* sequences are indicated with red asterisks. Branches for enhancins from betabaculoviruses and from alphabaculoviruses of *Lymantria* spp. and *Mamestra* spp. hosts are collapsed.

The top match of a BLASTx query with ORF69 was with an enhancin encoded by *Choristoneura fumiferana* multiple nucleopolyhedrovirus (CfMNPV [47]; GenBank accession no. NP_848341). Enhancins are zinc metalloproteases encoded by some alpha- and betabaculoviruses as well as bacteria [48]. The enhancins have been shown to promote primary infection of the host midgut epithelium by degrading proteins of the peritrophic matrix, which acts as a protective barrier [49,50,51], though some results suggest an additional or alternative mechanism for its effects [52,53]. Phylogenetic inference of the relationships among ORF69 and other enhancins grouped the ORF69 sequence with the enhancins from group I *Choristoneura* spp. alphabaculoviruses, including CfMNPV, *Choristoneura occidentalis* nucleopolyhedrovirus BC_1 (ChocNPV-BC_1) and *Choristoneura rosaceana* nucleopolyhedrovirus NB_1 (ChroNPV-NB_1) [54] (Figure 5). ORF69 and the *Choristoneura* spp. *Alphabaculovirus* enhancins grouped with a larger set of enhancins from alphabaculoviruses of hosts from subfamily Noctuinae. Other group I *Alphabaculovirus* enhancin genes were distributed among group II *Alphabaculovirus* sequences in a separate clade that included the *vef-1* and *vef-2* enhancin genes from viruses of *Lymantria* spp. hosts.

Metalloproteases are characterized by a zinc-binding motif, HEXXH, where X is any amino acid. The histidines in this motif coordinate a zinc ion that is essential for proteolytic activity, while the glutamate residue catalyzes the hydrolysis of the substrate peptide bond [55]. An alignment of ORF69 with 89 additional baculovirus enhancin sequences yielded a consensus zinc-binding motif sequence of HEIGH. However, the sequence of this motif in PastNPV ORF69 was found to be REIGH, with an arginine replacing the first histidine in the motif. Some of the *Alphabaculovirus* and betabaculovirus enhancin sequences deviate from the consensus zinc-binding motif to an even greater extent [48,56], and it is not clear if these ORFs or ORF69 encode an active metalloprotease.

PastNPV ORF75 shared the highest degree of sequence identity with LoobMNPV ORF78. Homologs of this ORF were also identified in a mixture of alphabaculoviruses and betabaculoviruses by BLASTx. An HHpred query with a multiple sequence alignment of 24 ORF75 homologs only yielded one reasonably convincing match with the nonstructural protein NS3 of Diatraea saccharalis densovirus (GenBank accession no. NP_046812.1), with a probability of 90.07%. The NS3 gene of the related Junonia coenia densovirus was shown to be required for densovirus DNA replication [57].

The predicted amino acid sequence of ORF126 shares 51.9% sequence identity with ORF141 of Antheraea pernyi nucleopolyhedrovirus (AnpeNPV, strain Liaoning; [58]). Homologs of this ORF are also found in other isolates of *Antherea* sp. NPVs and related NPVs from other saturniid hosts, as well as isolates of *Choristoneura* NPVs, Anticarsia gemmalis multiple nucleopolyhedrovirus (AgMNPV; [59]), Neophasia sp. nucleopolyhedrovirus (NespNPV, GenBank accession no. MK293724), and Epiphyas postvittana nucleopolyhedrovirus (EppoNPV; [60]). No homologs of this ORF were detected in group II *Alphabaculovirus* or betabaculovirus genomes, and no convincing matches were obtained from an HHpred query with an alignment of 19 ORF126 homologs. In a phylogeny inferred from an alignment of ORF126 and related sequences, ORF126 was placed in a position basal to the other homologs of this ORF (Figure S3).

#### 3.3.2. ORFs Not Found in Other Baculovirus Genomes

Six ORFs annotated for PastNPV-473 were not found to have homologs in any other baculovirus sequence available in GenBank (Table S2). Promoter motifs associated with both early and late baculovirus gene expression were identified for all six ORFs (Table 1). Queries with four of these ORFs (15, 27, 92, and 104) using BLASTx and HHpred did not return any matches with significant sequence similarity. A BLASTx query with ORF10 returned a top match with a hypothetical protein of the bacterium *Chitinophaga caeni* (GenBank accession no. WP_198405806) with 54.5% sequence identity and an e-value of 2.6 × 10^−20^. The predicted amino acid sequence of ORF10 contained eight complete copies and one partial copy of the sequence MAYVTDLS, which aligned with a similar repeat sequence in the *C. caeni* polypeptide.

**Figure 6:**
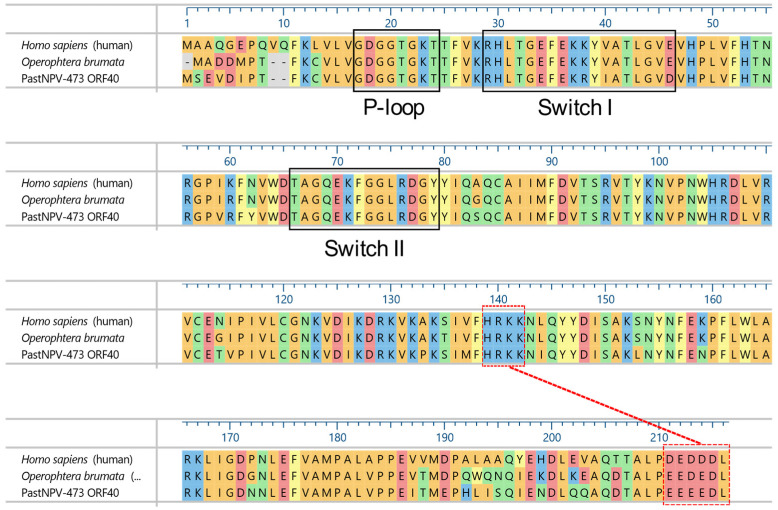
Amino acid sequence alignment of PastNPV-473 ORF40 with human and winter moth (*O. brumata*) homologs of Ran GTPase. Conserved P-loop, switch I, and switch II domains are indicated by black boxes, while a conserved acidic C-terminus (residues 211–216) and the basic patch with which it interacts (residues 139–142) are indicated by red dashed boxes and connecting line.

Queries with the ORF40 amino acid sequence indicated that it was a homolog of the Ran GTPase, a member of the Ras superfamily [61]. Ran regulates nucleocytoplasmic trafficking of macromolecules across the nuclear envelope and is involved in steps of the eukaryotic cell cycle, including mitotic spindle assembly and nuclear envelope formation [62]. The ORF40 polypeptide was found to share 84.0–88.3% sequence identity with Ran homologs from insects, with the highest BLASTx match being with the Ran GTPase of the navel orangeworm, *Amyelois transitella* (Lepidoptera: Pyralidae; GenBank accession no. XP_013194495). An examination of the alignment of the ORF40 amino acid sequence with other Ran sequences suggested that ORF40 encodes a full-length, functional Ran GTPase, with a conserved phosphate-binding loop (P-loop; GDGGTGKT) (Figure 6). The Ran Switch I and Switch II motifs, which undergo conformational changes in Ran when GDP is exchanged for GTP, were also present in ORF40, as was a conserved C-terminal acidic tail which interacts with a conserved basic sequence to stabilize the GDP-bound form of Ran [63,64].

## 4. Discussion

PastNPV is one of five group I alphabaculoviruses including LoobMNPV, CapoNPV, CyunNPV, and OxocNPV that can be distinguished from other group I alphabaculoviruses on the basis of branch lengths in phylograms and ORF synteny (Figure 3 and Figure 4). These viruses originate from a disparate group of host families including Crambidae (PastNPV), Pieridae (CapoNPV), Lasiocampidae (CyunNPV), Saturniidae (LoobMNPV), and Limacodidae (OxocNPV). A previous study failed to detect infectivity of PastNPV against larvae of seven other crambid species, three species of family Noctuidae, and one species of family Autostichidae [9]. We similarly were unable to detect infectivity of our sample of PastNPV when fed to neonate larvae of *Plutella xylostella* (diamondback moth, family Plutellidae) or *Trichoplusia ni* (cabbage looper, family Noctuidae), although we cannot exclude the possibility that our sample had lost infectivity during storage. Hence, the biological control potential of PastNPV may be limited to addressing outbreaks of *P. stagnalis* in rice.

The remaining 17 group I alphabaculoviruses of Figure 3 occur in two well-supported subclades, clade I.a and clade I.b. Clade I.a includes AcMNPV and other closely related alphabaculoviruses, and clade I.b consists of a more divergent group of alphabaculoviruses mostly from tree and forest pests, such as OpMNPV [65]). While Kimura-2-parameter nucleotide distances clearly indicate that PastNPV cannot be classified into any currently existing *Alphabaculovirus* species [40], the relationships of PastNPV and the four other more disparate viruses to the clade I.a and I.b alphabaculoviruses are unclear. The incongruence of the group I trees in Figure 3 could be due to the divergent nature of sequences from PastNPV and the other four viruses resulting in relatively long branch lengths, and relationships of these viruses with each other and the other group I alphabaculoviruses will likely require additional sequences from similar viruses to resolve.

The absence of a Ran GTPase homolog in other baculovirus genomes and the high degree of sequence identity between insect Ran GTPases and PastNPV ORF40 suggests that a virus of the PastNPV lineage had acquired a homolog of a cellular Ran sequence by horizontal gene transfer relatively recently. The preservation of motifs involved in Ran’s function in the ORF40 sequence further suggests that ORF40 encodes a functional Ran GTPase. Expression of the PastNPV homolog may affect egress of progeny PastNPV nucleocapsids from the host nucleus, or alter the nuclear or cellular mileau in a way that impacts DNA replication or virion assembly. The impact of expression of a Ran GTPase on baculovirus replication will require additional research.

## Figures and Tables

**Table 1:** Annotated ORFs unique to the PastNPV-473 genome.

ORF	Position (nt)	Amino Acids/kDa	Promoter Motifs ^1^	BLASTx Match
10	11799⟵12035	78/8.7	C, L	hypothetical protein [Chitinophaga caeni]
15	17875⟵18756	293/35.5	C, L, T	-
27	27159⟵27761	200/23.3	C	-
40	36077⟵36721	214/24.6	C	PREDICTED: GTP-binding nuclear protein Ran [Amyelois transitella]
92	85688⟵86476	262/31.6	C, L, T	-
104	95660⟵96394	244/29.2	C, T	-

^1^ Early (C, T) and late (L) gene promoter motifs identified within 200 bp of the start codon. C: CAKT; L: TAAG; T: TATAA.

## Data Availability

Sequence data reported in this study have been deposited in GenBank, accession no. ON704650.

## Supplementary Materials

The following supporting information can be downloaded at: https://www.mdpi.com/article/10.3390/v14102289/s1, Figure S1: Phylogeny of alphabaculoviruses in the group I clade; Figure S2: Alignment of consensus hr repeat sequences from Oxoc-435, AcMNPV-C6, BmNPV-T3, and PastNPV-473; Figure S3: Phylogeny of PastNPV-473 ORF126 homologs; Table S1: Viruses used for phylogenetic inference; Table S2: PastNPV-473 open reading frames (ORFs) and homologous repeat regions (*hrs*); Table S3: PastNPV-473 SNPs and indels; and Table S4: Kimura-2-parameter distances.
