# Supplementary material for: The Parapoynx Stagnalis Nucleopolyhedrovirus (PastNPV), a Divergent Member of the Alphabaculovirus Group I Clade, Encodes a Homolog of Ran GTPase
Source: Viruses. 2022 Oct 18;14(10):2289. doi: 10.3390/v14102289 (PMC9610796; doi:10.3390/v14102289)
Supplement: Supplementary file 1 [file viruses-14-02289-s001.zip › Supplementary Figures.pdf]

Supplementary Figures

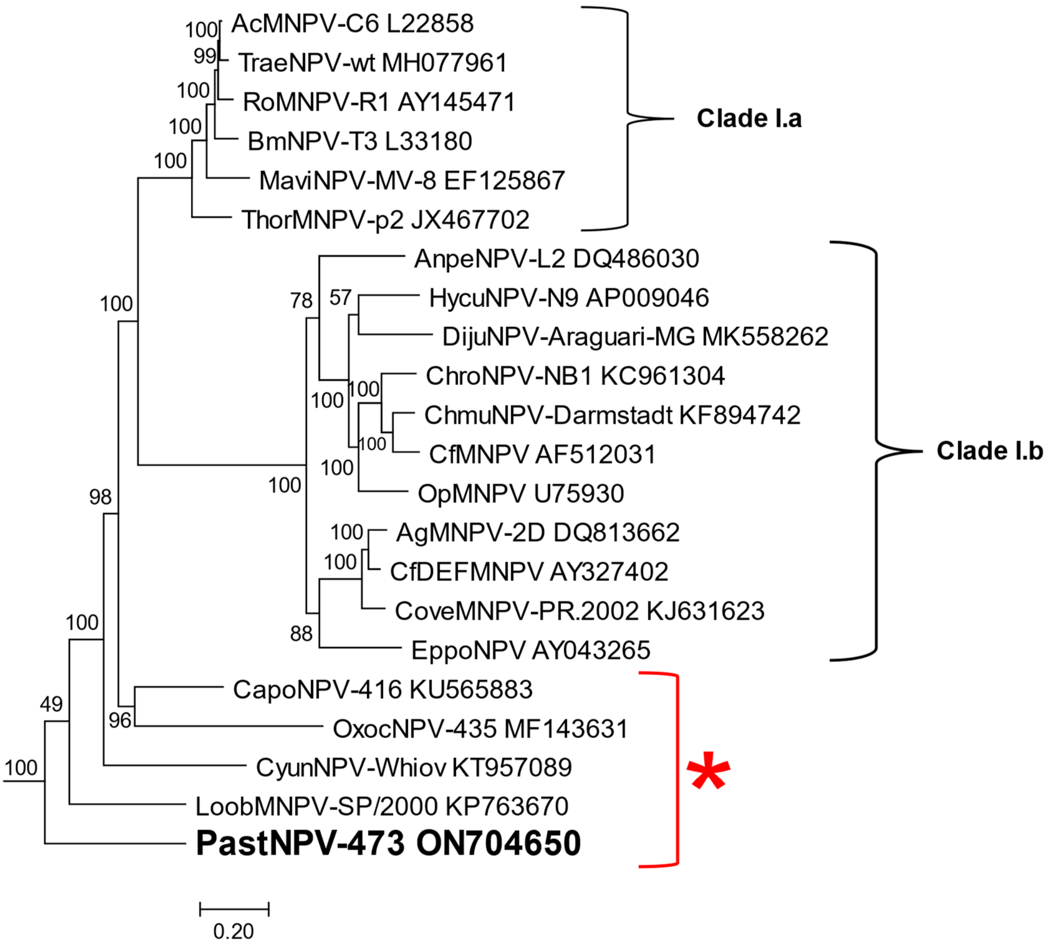

Figure S1. Phylogeny of alphabaculoviruses in the group I clade. An ML phylogram inferred from concatenated MUSCLE alignments of baculovirus core genes by maximum likelihood is shown, with bootstrap support for the branches. Abbreviations and Genbank accession numbers of viruses in the tree are provided; the viruses used in this analysis are listed in Table S1. Clade I.a and I.b are indicated by brackets, and a red bracket and asterisk denotes a group of five alphabaculoviruses with variable placement in the group I subtree. PastNPV-473 is indicated in bold type.

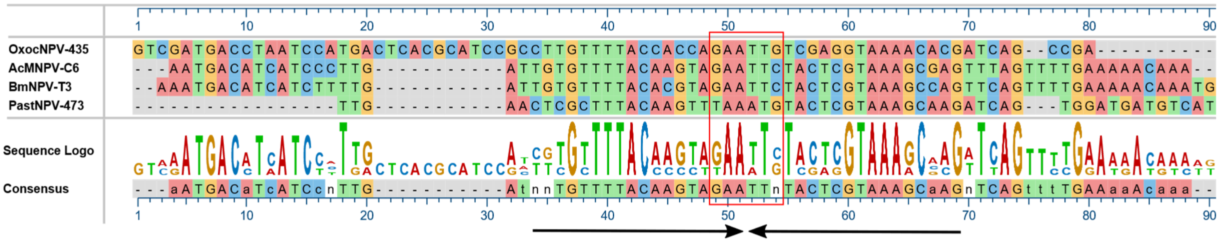

Figure S2. Alignment of consensus *hr* repeat sequences from Oxoc-435, AcMNPV-C6, BmNPV-T3, and PastNPV-473. The consensus sequence and logo generated from this alignment are as described for Figure 2. Arrows denote a conserved palindrome, and a red box encloses the center of the palindrome with the *Eco*R I restriction endonuclease site that is characteristic of the AcMNPV *hr*s.

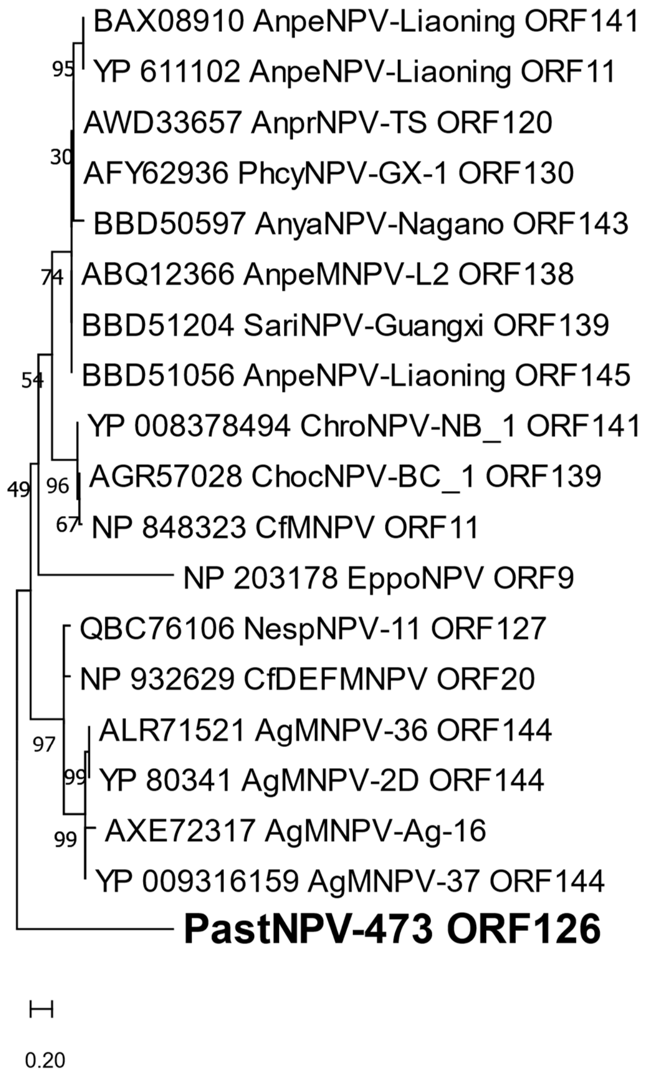

Figure S3. Phylogeny of PastNPV-473 ORF126 homologs. An ML phylogram inferred from concatenated MUSCLE alignments of PastNPV-473 ORF126 and related sequences by maximum likelihood is shown, with bootstrap support for the branches. Abbreviations and Genbank accession numbers of viruses in the tree are provided; the viruses used in this analysis are listed in Table S1. PastNPV-473 is indicated in bold type.
