# Supplementary material for: The Parapoynx Stagnalis Nucleopolyhedrovirus (PastNPV), a Divergent Member of the Alphabaculovirus Group I Clade, Encodes a Homolog of Ran GTPase
Source: Viruses. 2022 Oct 18;14(10):2289. doi: 10.3390/v14102289 (PMC9610796; doi:10.3390/v14102289)
Supplement: Supplementary file 1 [file viruses-14-02289-s001.zip › Table S2.pdf]

Table S2. PastNPV-473 open reading frames (ORFs) and homologous repeat regions (*hr*s)

ORF	Name	Position	aa	Top BLASTx match	% identity (range of alignment)	Notes/AcMNPV homologues
**1**	*polh*	1→747	248	ph protein [Thysanoplusia orichalcea nucleopolyhedrovirus]	93.1 (231/248)	*ac8*
**2**	*pp78/83*	744←2426	560	pp78/83 [Oxyplax ochracea nucleopolyhedrovirus]	30.2 (173/573)	*ac9*
**3**	*pk-1*	2433→3242	269	Protein kinase-1 [Lonomia obliqua multiple nucleopolyhedrovirus]	63.8 (171/268)	*ac10*
**4**	*pe38*	3460←4599	379	PE38 [Lonomia obliqua multiple nucleopolyhedrovirus]	43.6 (44/101)	*ac153*
**5**	*ie-2*	5007→6239	410	RING-type E3 ubiquitin transferase IE2 [Rachiplusia ou MNPV (strain R1)]	32.8 (86/262)	*ac151*
	hr1	6528 - 6648	-	-	-	*2 repeats*
**6**	*p26*	6751→7482	243	P26 [Lonomia obliqua multiple nucleopolyhedrovirus]	54.2 (130/240)	*ac136*
**7**	*p10*	7530→7769	79	p10 protein [Bombyx mori nucleopolyhedrovirus] (isolate GXLuoC2)	53.6 (37/69)	*ac137*
**8**	*p74*	7934←9874	646	p74 [Plutella xylostella multiple nucleopolyhedrovirus]	82.0 (530/646)	*ac138*
**9**	*me53*	10003←11415	470	ME53 [Lonomia obliqua multiple nucleopolyhedrovirus]	46.2 (192/416)	*ac139*
**10**		11799←12035	78	hypothetical protein [Chitinophaga caeni]	54.6% (42/77)	8.75 repeats of the sequence MAYVTDLS
**11**	*gp64*	12145→13674	509	gp64 [Cyclophragma undans nucleopolyhedrovirus]	72.3% (366/506)	*ac128*
	hr2	13985 – 14377	-	-	-	6 repeats
**12**	*odv-e56*	14458→15567	369	ODV-E56 [Plutella xylostella multiple nucleopolyhedrovirus]	69.6% (240/345)	*ac148*
**13**	*ie-1*	15465←17282	605	early gene transactivator [Autographa californica nucleopolyhedrovirus] (isolate C6)	49.4% (295/597)	*ac147*
**14**	*ep23*	17381→17977	198	AcOrf-146 peptide [Autographa californica nucleopolyhedrovirus] (isolate C6)	56.9% (115/202)	*ac146*
**15**		17875←18756	293	-	-	
**16**		18800←19081	93	cyun12 [Cyclophragma undans nucleopolyhedrovirus]	58.6% (56/95)	*ac145*

**17**	*odv-ec27*	19078←19995	305	ORF_14 [Catopsilia pomona nucleopolyhedrovirus]	66.4% (209/315)	*ac144*
**18**	*odv-e18*	20020←20286	88	ODV-E18 [Maruca vitrata nucleopolyhedrovirus]	77.5% (69/89)	*ac143*
**19**	*p49*	20283←21707	474	P49, 49K [Lonomia obliqua multiple nucleopolyhedrovirus]	70.8% (334/472)	*ac142*
**20**	*exon0*	21736←22539	267	IE-0 [Lonomia obliqua multiple nucleopolyhedrovirus]	51.2% (133/260)	*ac141*
**21**	*p24*	22851→23459	202	p24 capsid protein [Plutella xylostella multiple nucleopolyhedrovirus]	56.9% (111/195)	*ac129*
**22**	*ac78-like*	23470→23811	113	-	-	
**23**	*gp16*	23835→24143	102	gp16 protein [Thysanoplusia orichalcea nucleopolyhedrovirus]	77.5% (79/102)	*ac130*
**24**	*pp34*	24144→25235	363	PE protein [Thysanoplusia orichalcea nucleopolyhedrovirus]	49.6% (171/345)	*ac131*
**25**		25245→25823	192	hypothetical protein [Choristoneura rosaceana nucleopolyhedrovirus]	33.2% (68/205)	*ac132*
**26**	*alk-exo*	25867→27123	418	alk-exo [Catopsilia pomona nucleopolyhedrovirus]	52.0% (226/435)	*ac133*
**27**		27159←27761	200	-	-	
	hr3	27873 – 28207	-	-	-	*5 repeats*
**28**		28333→28587	84	Mv-ORF92 peptide [Maruca vitrata nucleopolyhedrovirus]	60.8% (48/79)	*ac120*
**29**		28684→29355	223	AcOrf-124 peptide [Autographa californica nucleopolyhedrovirus] (isolate C6)	38.0% (81/213)	*ac124*
**30**	*sod*	29534→29719	61	sod [Malacosoma neustria nucleopolyhedrovirus]	82.0% (50/61)	*ac31*
**31**	*pif-1*	29716←31311	531	Ac-PIF-1 [Autographa californica nucleopolyhedrovirus] (isolate E2)	75.7% (402/531)	*ac119*
**32**		31443←31730	95	unknown [Rachiplusia ou MNPV]	57.1% (52/91)	*ac117*
**33**	*bro-a*	31871←32065	64	DekiORF38 [Dendrolimus kikuchii nucleopolyhedrovirus]	57.4% (39/68)	
**34**	*bro-b*	32068←32241	57	baculovirus repeated namegeneORF [Condylorrhiza vestigialis MNPV]	48.9% (23/47)	
**35**	*pif-3*	32324→32944	206	per os infectivity factor-3 [Maruca vitrata nucleopolyhedrovirus]	65.9% (135/205)	*ac115*

**36**	*parg*	32961→34268	435	AcOrf-114 peptide [Autographa californica nucleopolyhedrovirus] (isolate C6)	36.4% (158/434)	*ac114*
**37**	*pif-7*	34301→34468	55	pif-7 [Cyclophragma undans nucleopolyhedrovirus]	74.6% (41/55)	*ac110*
**38**	*odv-ec43*	34493→35635	380	ODV-EC43 [Lonomia obliqua multiple nucleopolyhedrovirus]	70.7% (272/385)	*ac109*
**39**		35653→35991	112	hypothetical protein [Thysanoplusia orichalcea nucleopolyhedrovirus]	57.3% (59/103)	*ac108*
**40**		36077←36721	214	PREDICTED: GTP-binding nuclear protein Ran [Amyelois transitella]	88.3% (189/214)	
	hr4	36896 - 37223	-	-	-	5 repeats
**41**		38032←37349	227	Mv-ORF83 peptide [Maruca vitrata nucleopolyhedrovirus]	78.8% (167/212)	*ac106/107*
**42**	*vp80*	38053←40056	667	vp80 [Troides aeacus nucleopolyhedrovirus]	43.3% (221/510)	*ac104*
**43**	*p45*	39866→41233	455	unknown [Rachiplusia ou MNPV]	76.0% (295/388)	*ac103*
**44**	*p12*	41214→41549	111	P12 [Dione juno nucleopolyhedrovirus]	45.5% (50/110)	*ac102*
**45**	*p40*	41563→42639	358	p40 [Plutella xylostella multiple nucleopolyhedrovirus]	67.8% (246/363)	*ac101*
**46**	*p6.9*	42698→42859	53	-	-	*ac100*
**47**	*lef-5*	43668←42856	270	Late expression factor 5 [Lonomia obliqua multiple nucleopolyhedrovirus]	62.5% (170/272)	*ac99*
**48**	*38k*	43606→44529	307	38K protein [Thysanoplusia orichalcea nucleopolyhedrovirus]	61.0% (188/308)	*ac98*
**49**	*pif-4*	44516←45037	173	pif-4 protein [Thysanoplusia orichalcea nucleopolyhedrovirus]	75.7% (131/173)	*ac96*
**50**	*dnahel*	45027→48668	1213	DNA helicase [Lonomia obliqua multiple nucleopolyhedrovirus]	68.4% (849/1241)	*ac95*
**51**	*odv-e25*	48701←49387	228	odv-e25 [Cyclophragma undans nucleopolyhedrovirus]	72.4% (165/228)	*ac94*
**52**	*p18*	49399←49872	157	p18 [Catopsilia pomona nucleopolyhedrovirus]	77.7% (122/157)	*ac93*
**53**	*p33*	49871→50647	258	AcOrf-92 peptide [Autographa californica nucleopolyhedrovirus] (isolate C6)	64.3% (166/258)	*ac92*

**54**		50668←51180	170	ORF152 [Leucania separata nucleopolyhedrovirus]	41.6% (42/101)	
**55**	*lef-4*	51397←52788	463	late expression factor 4 [Bombyx mori nucleopolyhedrovirus] (isolate Brazilian)	63.3% (295/466)	*ac90*
**56**	*vp39*	52812→53882	356	VP39 [Lonomia obliqua multiple nucleopolyhedrovirus]	67.4% (242/359)	*ac89*
**57**	*cg30*	53938→54687	249	CG30 [Lonomia obliqua multiple nucleopolyhedrovirus]	44.8% (117/261)	*ac88*
**58**	*vp91*	54689←57211	840	viral capsid associated protein [Autographa californica nucleopolyhedrovirus] (isolate C6)	64.9% (550/847)	*ac83*
**59**	*tlp20*	57180→57698	172	TELOKIN-like protein [Lonomia obliqua multiple nucleopolyhedrovirus]	40.2% (82/204)	*ac82*
**60**	*ac81*	57559→58242	227	hypothetical protein [Lonomia obliqua multiple nucleopolyhedrovirus]	78.7% (174/221)	*ac81*
**61**	*gp41*	58235→59274	379	GP41 [Lonomia obliqua multiple nucleopolyhedrovirus]	70.8% (259/366)	*ac80*
**62**		59384→59698	104	hypothetical protein [Thysanoplusia orichalcea nucleopolyhedrovirus]	67.3% (70/104)	*ac79*
**63**		59704→60018	104	orf78 [Bombyx mori nucleopolyhedrovirus] (isolate C1)	54.9% (56/102)	*ac78*
**64**	*vlf-1*	60031→61170	379	vlf-1 [Troides aeacus nucleopolyhedrovirus]	83.0% (313/377)	*ac77*
**65**		61198→61452	84	hypothetical protein [Lonomia obliqua multiple nucleopolyhedrovirus]	77.4% (65/84)	*ac76*
**66**		61463→61864	133	hypothetical protein [Lonomia obliqua multiple nucleopolyhedrovirus]	72.9% (97/133)	*ac75*
**67**		61879→62415	178	ac74 [Oxyplax ochracea nucleopolyhedrovirus]	48.0% (86/179)	*ac74*
**68**	*iap-2*	63130←62420	236	Inhibitor of apoptosis 2 [Lonomia obliqua multiple nucleopolyhedrovirus]	55.1% (146/265)	*ac71*
**69**	*vef*	63154→65337	727	enhancin-like [Choristoneura fumiferana multiple nucleopolyhedrovirus]	47.9% (342/714)	
**70**	*mtase*	65326←66108	260	AcMNPV orf69 [Bombyx mori nucleopolyhedrovirus] (isolate T3)	58.1% (151/260)	*ac69*
**71**	*pif-6*	66484←66089	131	Orf-68 [Autographa californica nucleopolyhedrovirus] (isolate BAC10:KO1629)	71.1% (91/128)	*ac68*

**72**	*lef-3*	66493→67644	383	LEF-3 [Rachiplusia ou MNPV]	48.8% (189/387)	*ac67*
**73**	*desmop*	67633←69777	714	Desmoplakin [Lonomia obliqua multiple nucleopolyhedrovirus]	34.0% (269/792)	*ac66*
**74**	*dnapol*	69795→72755	986	DNA polymerase [Lonomia obliqua multiple nucleopolyhedrovirus]	67.3% (680/1011)	*ac65*
	hr5	73049 - 73369	-	-	-	5 repeats
**75**		73445←73909	154	hypothetical protein [Lonomia obliqua multiple nucleopolyhedrovirus]	36.0% (59/164)	
**76**	*gp37*	73936→74859	307	GP37 protein [Choristoneura diversana nucleopolyhedrovirus]	71.0% (218/307)	*ac64*
**77**		75037←75516	159	hypothetical protein [Lonomia obliqua multiple nucleopolyhedrovirus]	45.2% (70/155)	*ac63*
**78**	*lef-9*	75566←77071	501	late expression factor-9 [Plutella xylostella multiple nucleopolyhedrovirus]	83.1% (412/496)	*ac62*
**79**	*fp25k*	77151→77777	208	fp25k [Catopsilia pomona nucleopolyhedrovirus]	76.7% (155/202)	*ac61*
**80**	*chaB2*	77844→78113	89	ChaB-like protein [Oxyplax ochracea nucleopolyhedrovirus]	64.0% (48/75)	*ac60*
**81**	*chaB1*	78110→78619	169	unknown [Plutella xylostella multiple nucleopolyhedrovirus]	47.0% (85/181)	*ac58/59*
**82**		78647←78874	75	hypothetical protein [Thysanoplusia orichalcea nucleopolyhedrovirus]	40.0% (28/70)	*ac56*
**83**		78874←79116	80	cyun96 [Cyclophragma undans nucleopolyhedrovirus]	50.0% (40/80)	*ac55*
**84**	*vp1054*	79219←80292	357	VP1054 [Lonomia obliqua multiple nucleopolyhedrovirus]	64.6% (239/370)	*ac54*
**85**	*lef-10*	80147←80386	79	late expression factor-10 [Maruca vitrata nucleopolyhedrovirus]	66.2% (47/71)	*ac53a*
**86**	*U-box/RING-like domain protein*	80392←80805	137	hypothetical protein [Thysanoplusia orichalcea nucleopolyhedrovirus]	70.6% (96/136)	*ac53*
**87**		80807→81388	193	unknown [Rachiplusia ou MNPV]	45.0% (89/198)	*ac52*
**88**	*bjdp*	81377←82414	345	hypothetical protein [Lonomia obliqua multiple nucleopolyhedrovirus]	36.7% (119/324)	*ac51*

**89**	*lef-8*	82441→85068	875	lef8 [Catopsilia pomona nucleopolyhedrovirus]	71.4% (649/909)	*ac50*
**90**		85085←85492	135	AcMNPV orf44 [Bombyx mori nucleopolyhedrovirus] (isolate T3)	61.2% (79/129)	*ac44*
**91**		85473←85655	60	ORF-95 [Catopsilia pomona nucleopolyhedrovirus]	63.6% (35/55)	*ac43*
**92**		85688←86476	262	-	-	
**93**	*gta*	86563←88092	509	gta [Bombyx mori nucleopolyhedrovirus] (isolate My)	54.7% (281/514)	*ac42*
**94**	*lef-12*	88073←88678	201	late expression factor 12 [Bombyx mori nucleopolyhedrovirus]	56.1% (97/173)	*ac41*
**95**	*p47*	88677→89867	396	transcription regulator [Autographa californica nucleopolyhedrovirus] (isolate C6)	69.6% (275/395)	*ac40*
**96**	*adprase*	89901→90620	239	unknown [Rachiplusia ou MNPV]	69.2% (153/221)	*ac38*
**97**	*lef-11*	90520→90849	109	Late expression factor 11 [Lonomia obliqua multiple nucleopolyhedrovirus]	60.0% (57/95)	*ac37*
**98**	*pp31/39 k*	90843→91658	271	39K [Bombyx mori nucleopolyhedrovirus]	49.8% (141/283)	*ac36*
**99**	*v-ubi*	91712←91945	77	viral ubiquitin [Lonomia obliqua multiple nucleopolyhedrovirus]	89.6% (69/77)	*ac35*
**100**		91977→92630	217	ac34 [Troides aeacus nucleopolyhedrovirus]	47.7% (104/218)	*ac34*
**101**	*fgf*	92692→93240	182	fgf protein [Thysanoplusia orichalcea nucleopolyhedrovirus]	45.7% (80/175)	*ac32*
	hr6	93311 - 93607	-	-	-	*4 repeats*
**102**		93694→95091	465	ac30 [Troides aeacus nucleopolyhedrovirus]	51.6% (238/461)	*ac30*
**103**		95150→95362	70	Mv-ORF20 peptide [Maruca vitrata nucleopolyhedrovirus]	70.0% (49/70)	*ac29*
**104**		95660←96394	244	-	-	
**105**	*lef-6*	96458←97024	188	late expression factor-6 [Maruca vitrata nucleopolyhedrovirus]	76.4% (133/174)	*ac28*
**106**	*iap-1*	97036←97899	287	inhibitor of apoptosis-1 [Maruca vitrata nucleopolyhedrovirus]	64.6% (177/274)	*ac27*
**107**		97905←98285	126	hypothetical protein [Thysanoplusia orichalcea nucleopolyhedrovirus]	58.3% (70/120)	*ac26*

**108**	*dbp*	98292→99212	306	dbp [Catopsilia pomona nucleopolyhedrovirus]	70.0% (203/290)	*ac25*
**109**		99438←99773	111	hypothetical protein AGNV_104 [Anticarsia gemmatalis nucleopolyhedrovirus] (isolate 2D)	40.9% (45/110)	*ac57*
**110**	*pkip*	100045→100533	162	pkip [Troides aeacus nucleopolyhedrovirus]	46.1% (77/167)	*ac24*
**111**	*efp; f protein*	100591←102576	661	fusion protein [Rachiplusia ou MNPV]	32.9% (205/624)	*ac23*
**112**	*pif-2*	102662←103810	382	AcOrf-22 peptide [Autographa californica nucleopolyhedrovirus] (isolate C6)	84.8% (324/382)	*ac22*
**113**	*arif-1*	103848→104933	361	actin rearrangement inducing factor - 1 [Rachiplusia ou MNPV]	36.5% (111/304)	*ac20/21*
**114**		104965←105303	112	ORF-119 [Catopsilia pomona nucleopolyhedrovirus]	56.1% (46/82)	*ac19*
**115**		105311→106375	354	unknown [Rachiplusia ou MNPV]	43.2% (153/354)	*ac18*
**116**	*bv-ec31*	106405←107064	219	AcMNPV orf17 [Bombyx mori nucleopolyhedrovirus] (isolate T3)	52.1% (114/219)	*ac17*
**117**	*bv/odv-e26*	107033←107752	239	odv-e26 [Bombyx mori nucleopolyhedrovirus] (isolate My)	50.0% (111/222)	*ac16*
**118**	*egt*	107889←109391	500	UDP-glucosyl transferase [Bombyx mori nucleopolyhedrovirus] (isolate Thailand)	61.6% (310/503)	*ac15*
**119**	*lef-1*	109510→110256	248	LEF-1 [Bombyx mori nucleopolyhedrovirus] (isolate T3)	64.3% (153/238)	*ac14*
**120**	*38.7k*	110211→111176	321	hypothetical protein [Bombyx mori nucleopolyhedrovirus] (isolate H4)	54.7% (181/331)	*ac13*
**121**		111263→112276	337	hypothetical protein [Bombyx mori nucleopolyhedrovirus]	47.2% (161/341)	*ac11*
**122**	*ptp*	112321→112842	173	PTP [Bombyx mori nucleopolyhedrovirus] (isolate T3)	60.7% (102/168)	*ac1*
**123**		112817←113257	146	hypothetical protein [Thysanoplusia orichalcea nucleopolyhedrovirus]	56.3% (81/144)	*ac4*
**124**		113305→113631	108	hypothetical protein [Lonomia obliqua multiple nucleopolyhedrovirus]	42.6% (52/122)	*ac5*
**125**	*lef-2*	113606→114220	204	late expression factor-2 [Maruca vitrata nucleopolyhedrovirus]	61.3% (122/199)	*ac6*

**126**		114223←114708	161	hypothetical protein [Antheraea pernyi nucleopolyhedrovirus] (isolate Liaoning)	51.9% (28/54)	
